# Resolution-enhanced Fourier ptychographic microscopy based on high-numerical-aperture illuminations

**DOI:** 10.1038/s41598-017-01346-7

**Published:** 2017-04-26

**Authors:** Jiasong Sun, Chao Zuo, Liang Zhang, Qian Chen

**Affiliations:** 1 Smart Computational Imaging (SCI) Laboratory, Nanjing University of Science and Technology, Nanjing, Jiangsu Province 210094 China; 2 Jiangsu Key Laboratory of Spectral Imaging & Intelligent Sense, Nanjing University of Science and Technology, Nanjing, Jiangsu Province 210094 China

## Abstract

High-resolution and wide field-of-view (FOV) microscopic imaging plays a central role in diverse applications such as high-throughput screening and digital pathology. However, conventional microscopes face inherent trade-offs between the spatial resolution and FOV, which are fundamental limited by the space-bandwidth product (SBP) of the optical system. The resolution-FOV tradeoff can be effectively decoupled in Fourier ptychography microscopy (FPM), however, to date, the effective imaging NA achievable with a typical FPM system is still limited to the range of 0.4–0.7. Herein, we report, for the first time, a high-NA illumination based resolution-enhanced FPM (REFPM) platform, in which a LED-array-based digital oil-immersion condenser is used to create high-angle programmable plane-wave illuminations, endowing a 10×, 0.4 NA objective lens with final effective imaging performance of 1.6 NA. With REFPM, we present the highest-resolution results with a unprecedented half-pitch resolution of 154 nm at a wavelength of 435 nm across a wide FOV of 2.34 mm^2^, corresponding to an SBP of 98.5 megapixels (~50 times higher than that of the conventional incoherent microscope with the same resolution). Our work provides an important step of FPM towards high-resolution large-NA imaging applications, generating comparable resolution performance but significantly broadening the FOV of conventional oil-immersion microscopes.

## Introduction

High-resolution wide-field imaging is essential for various applications in different fields, such as biological, biomedical research and digital pathology, which require large space-bandwidth product (SBP) to provide computational and statistical analyses for thousands of cells simultaneously across a wide field-of-view (FOV)^1–4^. However, conventional microscopes are always beset by the inherent trade-offs between the spatial resolution and FOV, limiting their SBPs and application areas. In the last decades, an enormous amount of research and considerable effort has been devoted to creating high-resolution wide-field microscopy techniques, like mechanical scanning and stitching, synthetic aperture microscopy, lens-free on-chip super-resolution technique, and Fourier ptychography microscopy (FPM)^5–13^. FPM is a recently developed technique that overcomes the physical SBP limit of a bright-field microscope by transforming the challenge into which can be solved through computation^13, 14^. Instead of starting with high resolution and stitching together a larger FOV, FPM uses low NA objective lens to take advantage of its innate large FOV and stitches together images in Fourier space to recover high resolution. Illumination angles are scanned sequentially with a programmable light-emitting diode (LED) array, shifting different amounts of high spatial frequency information into the low NA objective lens. This resolution improvement is analogous to coherent aperture synthesis^8, 9, 15–18^ and structured-illumination^5, 6, 19–22^ imaging. But distinct from synthetic aperture, FPM uses nonlinear optimization algorithms^13, 23–25^ similar to translational diversity^26, 27^ and ptychography^28–31^ to perform the reconstruction instead. Since the FOV is fixed in FPM and the final reconstruction resolution is determined by the sum of the objective lens and illumination NAs, the ratio of *NA*_*ill*_ (illumination NA) to *NA*_*obj*_ (objective NA) becomes the dominant factor for achieving higher improvement of SBP. To be more precisely, the theoretical enhancement of SBP just equals ${(1+\frac{N{A}_{ill}}{N{A}_{obj}})}^{2}$, explaining why most FPM platforms employ low NA objective lens with high illumination angles.

Although many significant progresses have been made in FPM for achieving higher data acquisition efficiency^24, 32–34^ and recovery accuracy^23, 34–39^ in the past few years, little is pursuing a larger SBP with high synthetic NA (*NA*_*syn*_) greater than unity. Since the illumination brightness, the angle of divergence for each LED element, and the flexibility of the commercial LED board are all limited by the processing technology, the illumination angles in most FPM systems have been limited to an NA of less than about 0.3–0.6. Thus the highest achievable half-pitch resolution for those reported FPM setups is usually constrained between 500–700 nm, which is far away from the need of recent high-resolution-imaging applications. In order to further improve the recovery resolution, instead of enlarging *NA*_*ill*_, a high-numerical-aperture FPM is implemented using high magnification objective lenses with large *NA*_*obj*_ to achieve the final *NA*_*syn*_ of 1.45^36^. However, since the reduction of FOV is much larger than the improvement of resolution in this FPM platform, the SBP is suppressed to some extent, making this FPM technique less appealing.

Herein, we introduce a resolution-enhanced FPM setup, termed REFPM, which utilizes an LED array and an oil-immersion condenser to significantly improve the recovery resolution of conventional FPM with an expanded *NA*_*ill*_ greater than unity, without sacrificing the FOV. In REFPM platform, the oil-immersion condenser collects the illumination lights from every LED elements and transfers them into plane-waves, achieving a striking *NA*_*ill*_ of 0.93 (dry) and 1.2 (oil). Although it seems convenient to directly expand the *NA*_*ill*_ by using an oil-immersion condenser, additional challenges have been brought about for achieving higher resolution accurately. Aiming to satisfy the sampling criteria for FPM in object and frequency space, we employ a denser LED array and carefully design our REFPM setup with proper system parameters, pushing the limit of resolving power of FPM while keeping the imaging field of view (FOV) uncompromised. Furthermore, in order to compensate the imperfections and uncertainties of the optical system, a series of computational correction methods are implemented in our iterative reconstruction algorithm. We evaluate the importance of each correction method experimentally and overcome those physical limitations and problems, such as pixel aliasing problem, illumination brightness inhomogeneity, and LED positional misalignment through computational correction. Finally, using 261 different illumination angles with a 10×/0.4 NA objective lens, we achieved an effective *NA*_*syn*_ of ~1.6 and a half-pitch resolution of 154 nm at a wavelength of 435 nm across a large FOV of 2.34 mm^2^, which constitutes a SBP of 98.7 megapixels. We also compare the performance of our REFPM setup against other commonly available high-NA microscopes, including conventional oil-immersion microscopes. The reconstructed results indicate that REFPM achieves a large SBP nearly 50 times higher than that of the conventional incoherent microscope with the same resolution. Therefore, this unique technique could not only generate comparable resolution performance but also significantly broaden the FOV of conventional oil-immersion microscopes, which would provide an important step of FPM towards high-resolution large-NA imaging applications.

## Results

### Resolution and SBP of different microscopy systems

**Figure 1:**
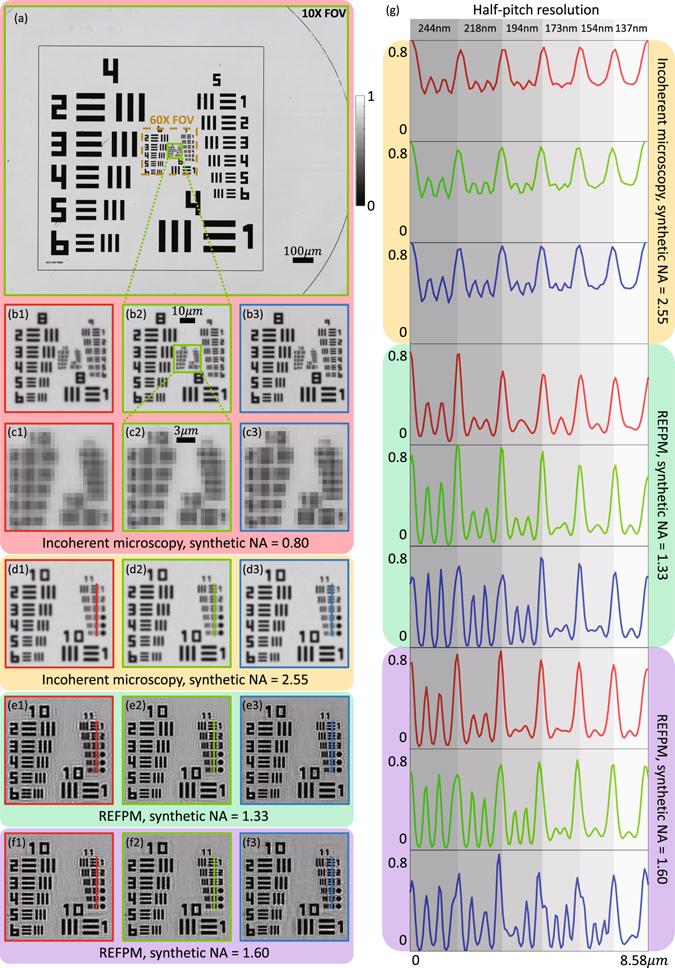
Imaging and recovery results of conventional bright-field microscopy and REFPM systems for the same USAF resolution target. (**a**) The full FOV of the incoherent microscopy image using a 10 × 0.4 NA objective lens with 0.4 NA illumination. (**b**,**c**) Enlarged sub-regions of Fig. 1(a) in R/G/B channels. (**d**) High-resolution incoherent microscopy results using a 60 × 1.35 NA objective lens with 1.2 NA illumination in three channels. (**e**,**f**) REFPM recovery results obtained by employing a 10 × 0.4 NA objective lens with 0.93 NA and 1.2 NA illumination in R/G/B channels. (**g**) Line-scan profiles from the selected position of targets in Fig. 1(d1–f3).

We evaluate the performance of the REFPM platform by reconstructing a resolution target. Figure 1 compares the resolution and SBP of conventional bright-field microscopy and REFPM systems for the same USAF resolution target. Figure 1(a) shows the full FOV of the incoherent microscopy image using a 10×/0.4 NA objective lens with 0.4 NA green light illumination. The windowed small region in Fig. 1(a) is enlarged and presented in Fig. 1(b2), while the same part of the microscopy image in red and blue channels are presented in Fig. 1(b1,b3). As can be seen, the highest distinguishable resolution target elements in Fig. 1(b1–b3) are Elements 5 in Group 9, limited by the imaging pixel-size $(\frac{6.5\mu m}{10}=650\,{\rm{nm}})$. We further enlarge the middle part of the resolution target to present patterns in Group 10 and Group 11 more clearly [Fig. 1(c1–c3)]. Since the imaging resolution is limited by the camera, all the patterns in Fig. 1(c1–c3) cannot be recognized. We also performed a set of simulations to validate how does this imaging pixel-size restrict the resolution (Supplementary Information A). Next, using a 60×/1.35 NA objective lens with 1.2 NA monochromatic illuminations, high-resolution incoherent microscopy results are obtained, as shown in Fig. 1(d1–d3). The line-scan profiles from the selected positions in Fig. 1(d1–d3) are given in top three rows of Fig. 1(g). By employing REFPM with a 10 × 0.4 NA objective lens and 0.93 NA monochromatic illuminations, intensity recovery results are obtained, as shown in Fig. 1(e1–e3). Here, we used the oil-immersion condenser but did not add the immersion oil between the sample and the condenser. Thus, the *NA*_*ill*_ could not surpass 1 in this condition. Comparing the theoretical (237 nm, 197 nm, 164 nm) and measured half-pitch resolutions (218 nm, 194 nm, 173 nm) in three channels, it indicates that this REFPM system could nearly achieve the theoretical *NA*_*syn*_ of 1.33 (dry). Next, we added the immersion oil between the target and the condenser for raising the *NA*_*ill*_ to 1.2. As can be seen in Fig. 1(f1–f3), comparing the theoretical (197 nm, 164 nm, 136 nm) and measured half-pitch resolutions (194 nm, 173 nm, 154 nm) in three channels, a *NA*_*syn*_ close to 1.6 (oil) is realized using this REFPM system. Table 1 summarizes the SBPs which are achieved using these four microscopy systems, and it is obvious that our REFPM platform is able to provide an SBP about 98.5 megapixels, which is nearly 50 times higher than that of the conventional bright-field microscope with the same resolution. In Supplementary Video 1, we show a zooming video of the full-FOV raw image captured with conventional incoherent microscope (*NA*_*obj*_ = 0.4, *NA*_*ill*_ = 0.4, *λ* = 435 nm) and the reconstructed full-FOV image using REFPM (*NA*_*obj*_ = 0.4, *NA*_*ill*_ = 1.2, *λ* = 435 nm).

**Table 1:** Comparison of the measured half-pitch resolution, FOV, and SBP that are achieved using four different microscopy systems.

Half-pitch resolution and SBP for microscopy systems (*λ* = 435 *nm*)								
	Objectieve lens	FOV (*mm* ^2^)	Illumination NA	Theoretical synthetic NA	Theoretical half-pitch resolution (*nm*)	Theoretical SBP (*megapixels*)	Measured half-pitch resolution (*nm*)	Measured SBP (*megapixels*)
Conventional incoherent microscopy	10 × 0.40 NA	2.34	0.40 NA	0.80 NA	332	21.2	615	6.19
	60 × 1.35 NA	0.06	1.20 NA	2.55 NA	104	6	173	2.17
REFPM	10 × 0.40 NA	2.34	0.93 NA	1.33 NA	164	87	173	78.2
	10 × 0.40 NA	2.34	1.20 NA	1.60 NA	136	125	154	98.7

### Effectiveness of the correction methods

**Figure 2:**
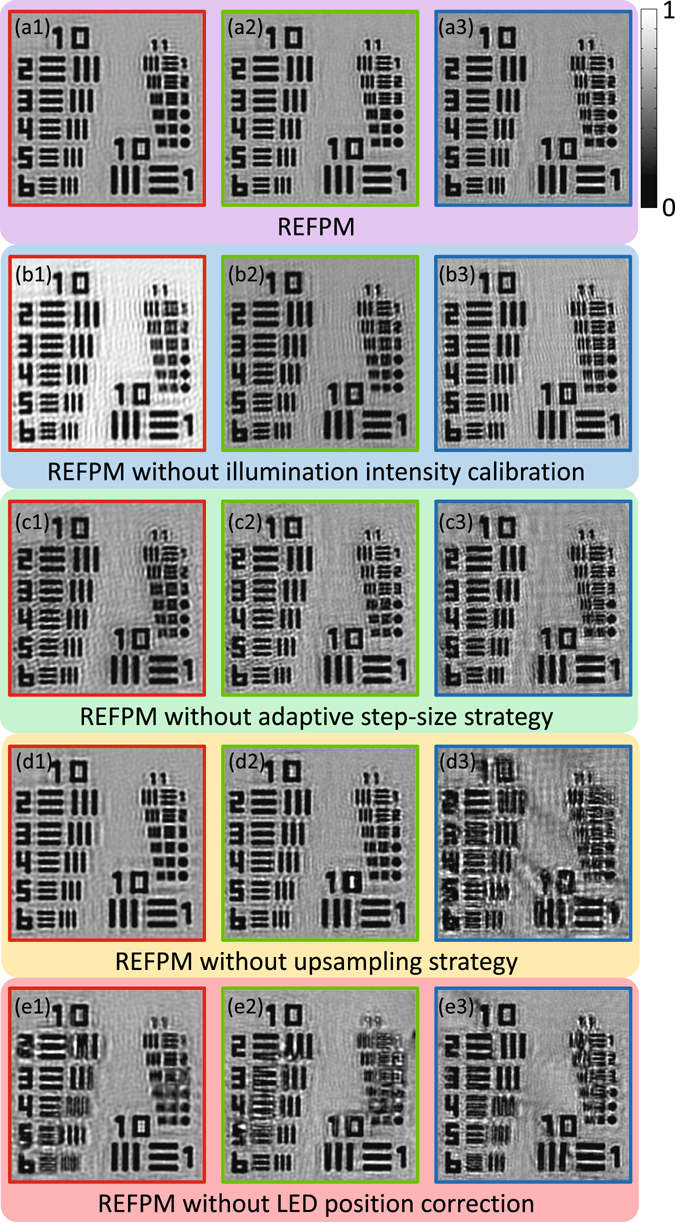
Comparison of the enlarged sub-regions of the measured USAF resolution target using different FPM reconstruction methods. (**a**) Reconstructed intensity images in R/G/B channels using REFPM with all the correction methods implemented in this work. (**b**–**e**) REFPM recovered results in three channels without using illumination brightness calibration, adaptive step-size strategy, upsampling strategy, or positional misalignment correction respectively.

To validate the effectiveness of those correction methods described below, Fig. 2 shows some enlarged sub-regions from the recovered images using different FPM reconstruction methods. All the reconstruction results are recovered from the same raw low-resolution images while having the same iterative times. The REFPM with all the correction methods provides the best reconstruction quality and its recovered intensity images in R/G/B three channels are illustrated in Fig. 2(a1–a3). Next, if we do not calibrate the illumination brightness before iterations, the resolution targets are deformed a bit, and a lot of mesh patterns will emerge in the background noticeably, as shown in Fig. 2(b1–b3). Then, by implementing the REFPM without adaptive step-size strategy, the edge of the resolution elements is distorted and the highest achievable resolutions in the R/G/B three channels decreased a little [Fig. 2(c1–c3)]. According to the sampling criterion for FPM in object space, when we use the red light to illuminate the object, Nyquist criterion is satisfied in the imaging system of our REFPM platform. But those low-resolution images in blue channel are suffering from serious pixel aliasing problem. Therefore, we got the same results in the red channel with [Fig. 2(a1)] or without [Fig. 2(d1)] using the upsampling strategy. But in the blue channel, reconstruction quality reduced significantly without the help of upsampling strategy [Fig. 2(d3)]. At last, as introduced in ref. 37, the recovery quality is very sensitive to the positional misalignment of the LED array. Thus, once we remove the LED position correction process from the REFPM algorithm, the reconstructed results would be extremely distorted and nearly none of the resolution elements in Group 11 is distinguishable [Fig. 2(e1–e3)]. Therefore, the significance of these correction methods has been illustrated clearly by comparing reconstruction results in Fig. 2. It indicates that the LED positional misalignment correction process is the pivotal point for not only achieving this unprecedented high resolution with low-NA objective lens, but relaxing the requirement of optical aligning. Moreover, if the Nyquist criterion is not satisfied and pixel aliasing problem occurs, the upsampled FPM method with sufficient data redundancy could still guarantee the recovery accuracy.

### Wide-FOV high-resolution microscopy of stained cell sample

**Figure 3:**
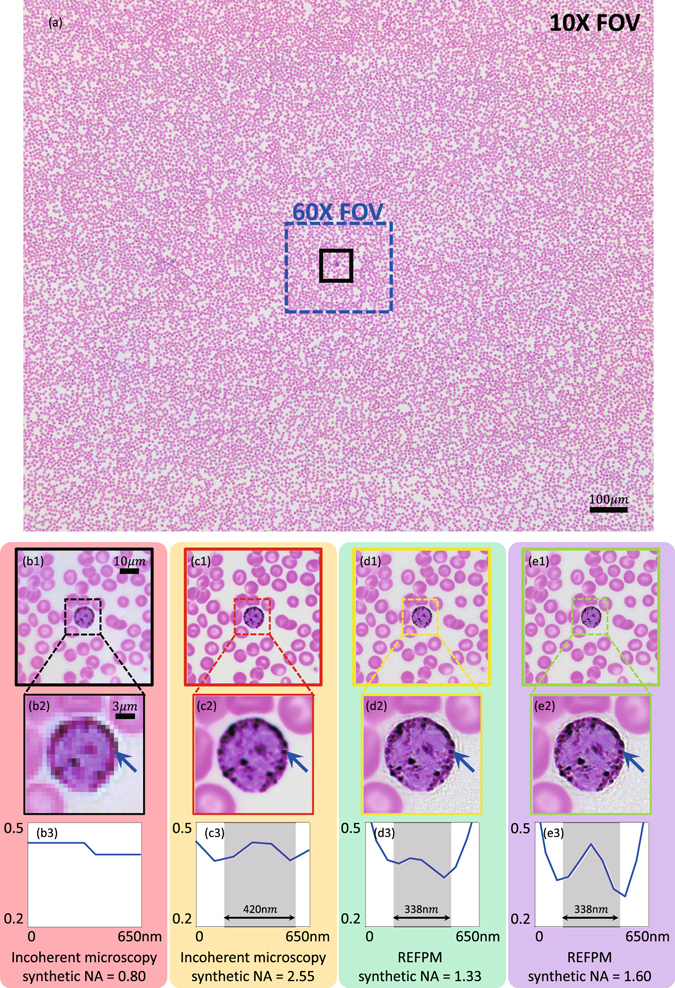
Imaging and recovery results of conventional bright-field microscopy and REFPM systems for the same human blood cell smear. (**a**) The full FOV of the incoherent microscopy image using a 10 × 0.4 NA objective lens with 0.4 NA illumination. (**b1**) Enlarged sub-regions of Fig. 3(a). (**c1**–**e1**) Imaging and recovery results of the same sub-region using conventional bright-field microscopy and REFPM with different *NA*_*syn*_. (**b2**–**e2**) Color images of a stained blood cell using four different microscopy methods. (**b3**–**e3**) Line-scan profiles of the two-point structure from the position pointed by arrows in Fig. 3(b2–e2).

**Figure 4:**
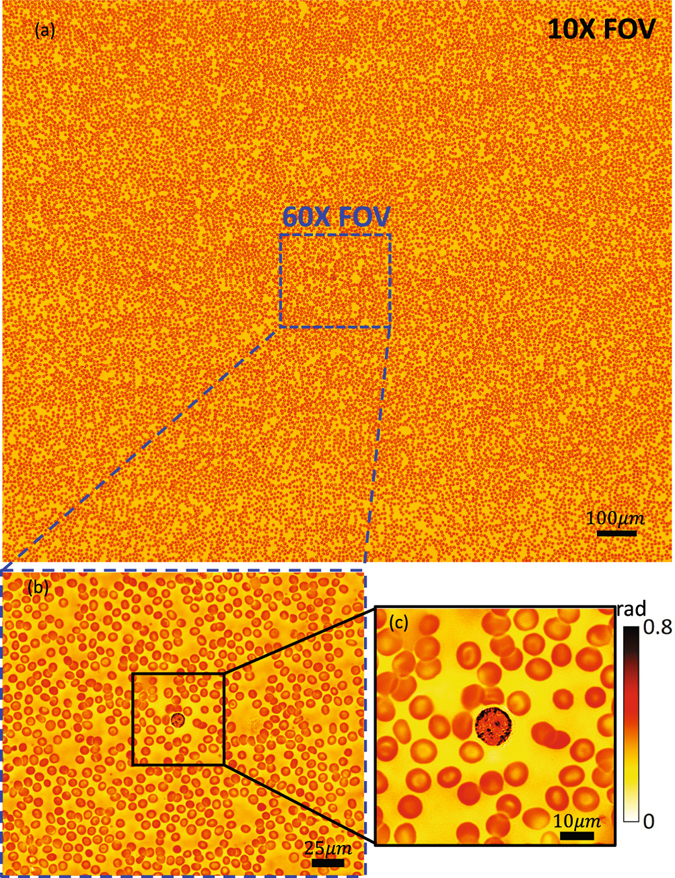
Recovered phase distribution of the blood cell smear using our REFPM platform. (**a**) The full FOV of the retrieved phase map using a 10 × 0.4 NA objective lens with 1.2 NA illumination. (**b**,**c**) Enlarged sub-regions of Fig. 4(a).

To obtain color images of the stained human blood cells via REFPM, we repeat the capture and reconstruction steps three separate times using red, green and blue LED illuminations from the same LED array, and then place each reconstruction in the appropriate color channel for the final color image in Fig. 3. We detail imaging performance in a selected image sub-regions, marked by black squares in Fig. 3(a). Images from the conventional color microscope setup, using the same 10× and 60× objective lenses as noted above, are shown in Fig. 3(b1,c1). Image clarity increases as the objective lens NA increases, but at a sacrifice of a smaller FOV (marked with blue dashed window in Fig. 3(a)). The same sub-regions from our 1.33 *NA*_*syn*_ and 1.6 *NA*_*syn*_ REFPM reconstructions are presented in Fig. 3(d1,e1). The REFPM recovered image with 1.6 *NA*_*syn*_ contains details that are not resolved in any of the other images. For example, a stained blood cell from Fig. 3(e1) is further zoomed in (Fig. 3(e2)), showing two-point structure (pointed by blue arrows) that are clearly resolved by 1.6 NA REFPM (Fig. 3(e3)). In comparison, for 60× oil-immersion microscope and the 1.33 NA REFPM, this two-point structure is vaguely resolved (Fig. 3(c3,d3)), while REFPM provides a better imaging quality with higher contrast ratio. Furthermore, the recovered phase distribution of this blood cell smear is illustrated in Fig. 4(a). This phase map is recovered with 1.6 *NA*_*syn*_ in the red channel and it is displayed in false color. The enlarged sub-regions of Fig. 4(a) are shown in Fig. 4(b,c) to illustrate the phase recovery quality of our REFPM system. In addition, our method also applies to unstained cells and a set of recovered phase maps can be found in Supplementary Information B.

## Discussion

In this paper, we report a high-NA illumination based resolution-enhanced FPM platform, named REFPM, to generate high-resolution large-SBP reconstructions from a series of low-resolution raw images with the help of a 1.2 NA oil-immersion condenser and a 0.4 NA objective lens. It is demonstrated that REFPM system can significantly improve the resolution and achieve the final effective imaging performance of 1.6 NA, corresponding to a half-pitch resolution of 154 nm with a FOV of 2.34 mm^2^ at a wavelength of 435 nm. The maximum achievable SBP of our microscope is about 98.5 megapixels, which is nearly 50 times higher than that of the conventional incoherent microscope with the same resolution. We also investigate the importance of a series of correction algorithms for overcoming the physical limitations and uncertainties of the optical system, such as illumination brightness inhomogeneity, imaging noise, LED positional misalignment, and pixel aliasing problem. Those high-quality reconstructed results demonstrate that REFPM not only extends the SBP of conventional microscopes, but also yields a significantly enhanced imaging resolution far beyond (up to 3–4 times) the coherent diffraction limit, which makes it highly appealing for those applications that require high-resolution and large-SBP at the same time.

## Materials and Methods

### Optical setup

**Figure 5:**
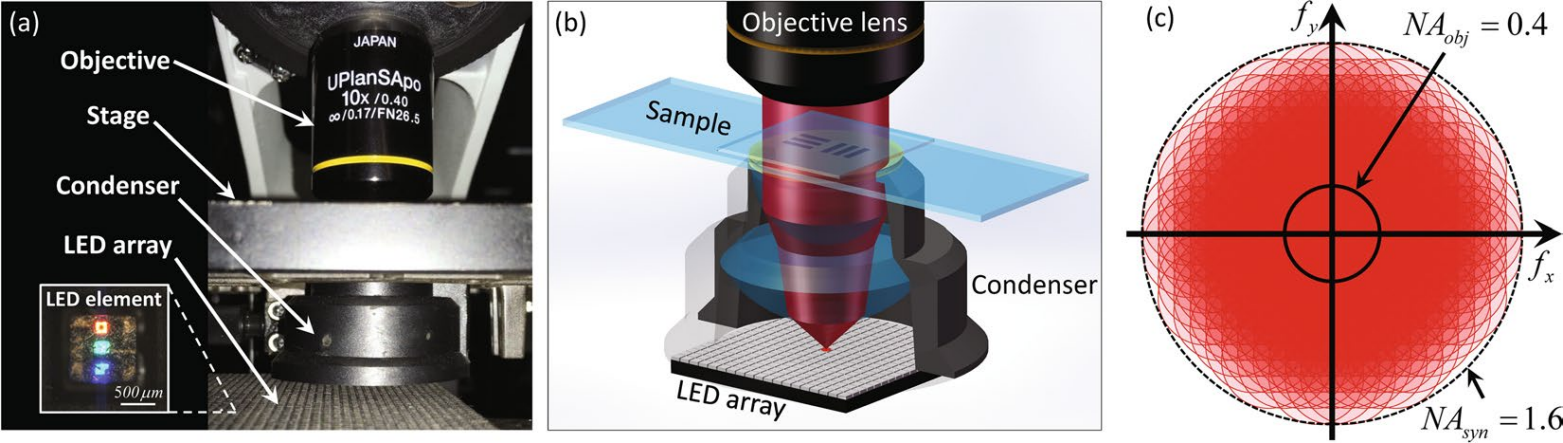
Optical setup of the LED array and condenser-based REFPM platform. (**a**) The experimental setup involves an LED array board, an oil-immersion condenser (1.20 NA) and an Olympus UPlanSApo 10×(0.40 NA) objective lens. (**b**) Schematic diagram of the illumination system in our REFPM platform. (**c**) Synthesized frequency apertures in the Fourier space using REFPM.

As depicted in Fig. 5, the REFPM setup in this paper consists of three major components: a programmable LED array, an oil-immersion condenser, and a microscopic imaging system. The commercial, multi-wavelength surface-mounted LED array (1.667 mm spacing) is placed at the front focal plane of the condenser to illuminate the specimen from different angles. The central wavelengths in R/G/B channels are 632 nm, 525 nm, 435 nm respectively, and the spectral linewidth at each wavelength is ~20 nm. During the imaging process, 261 LED elements on the board are lighted up sequentially and all of them are driven statically using a self-made LED controller board with an FPGA unit (EP4CE10E22C8N) to provide the logical control. The imaging system consists of two parts: a commercial bright-field microscope (CX22, Olympus, Japan) and a scientific CMOS camera (PCO.edge 5.5, 6.5 um pixel pitch). The camera is synchronized with the LED array by the same controller via two coaxial cables that provide the trigger and monitor the exposure status. We experimentally measure the system frame rate to be ~33 Hz for capturing full-frame (2560 × 2160) 16-bit images. Thus, all those 261 images are captured within 8 seconds. The data are transferred to the computer via a CameraLink interface.

### Sample preparation

The Extreme resolution target (Ready Optics Company, Calabasas, California, USA), 1951 USAF board from Group 4 to Group 11 (137 nm minimum spacing), is embedded in a standard microscope slide that suitable for oil-immersion objectives and oil-immersion condensers. The blood cell smear sample (Carolina Biological Supply Company, Burlington, North Carolina, USA) is prepared using normal (healthful) human blood and stained with hematoxylin and eosin^40^ to show general framework of crimson and white blood cells.

### Illumination brightness calibration

**Figure 6:**
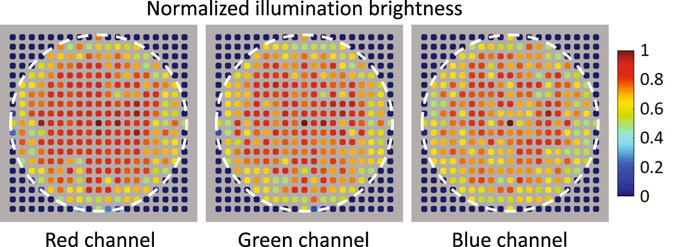
Normalized illumination brightness of every LED elements in R/G/B channels.

The illumination inconformity of the LED elements is one of the major systematic errors of FPM platforms. In this work, we implement the calibration procedure before measurement using an oil-immersion objective lens (60×/1.35 NA) with the oil-immersion condenser (1.20 NA), and fill the space between them with microscope oil (*n*_*oil*_ = 1.518). Since the NA of this objective lens is larger than that of the condenser, illumination lights from all the incident angles can be collected by the objective lens and recorded as bright-field images. First, we turn on the central LED in R/G/B channels sequentially and capture three monochrome images (**I**_1*R*_, **I**_1*G*_, **I**_1*B*_) with a same exposure time. Generally, the average intensity values of these monochrome images **I**_1*R*_,_1*G*,1*B*_ are quite different due to the brightness inconformity of the central LED in R/G/B channels. In order to balance the color of the LED array, we adjust the exposure times for the central LED in three channels to make sure that **I**_1*R*,1*G*,1*B*_ have the same average intensity value. Next, all the LED elements are lighted up sequentially and 261 images are captured in each channel, considering the average intensity of each image as the illumination brightness of each LED element. After obtaining the brightness of all the LEDs, we divide them by the brightness of the central LED to generate the normalized illumination brightness in three channels. These normalized parameters will be used as the intensity correction factors in the post-processing procedure. When a set of low-resolution images are captured, before iterative reconstruction, they need to be divided by their corresponding normalized illumination brightness to computationally compensate the illumination inconformity of the LED elements. Figure 6 displays the normalized illumination brightness of every LED elements in R/G/B channels. It is obvious that the illumination inconformity of the LED elements could be really huge (~50% maximum intensity difference), which need to be calibrated properly to achieve the expected recovery accuracy. Note that, those brightness values which are zeros denote that their corresponding LED elements are exceeding the NA of the condenser.

### Adaptive step-size strategy for FPM

Since the low-level of irradiance produces individual measurements that are heavily affected by noise in those dark-field images, we adopt the adaptive step-size strategy as introduced in ref. 39 to improve the stability and robustness of the reconstruction towards noise yet retain the fast initial convergence speed. In order to retrieve the pupil function of the objective lens, we implement the adaptive step-size strategy and an incremental gradient method^24^ with pupil function recovery procedure based on the following equations,

1 $${{\rm{\Psi }}}_{i}^{k}= {\mathcal F} \{\sqrt{\frac{{{\bf{I}}}_{i}}{{|{ {\mathcal F} }^{-1}\{{{\bf{P}}}_{i}^{k}{{\bf{O}}}_{i}^{k}\}|}^{2}}}{ {\mathcal F} }^{-1}\{{{\bf{P}}}_{i}^{k}{{\bf{O}}}_{i}^{k}\}\}$$

2 $${{\bf{O}}}_{i+1}^{k}={{\bf{O}}}_{i}^{k}-{\alpha }^{k}\frac{|{{\bf{P}}}_{i}^{k}|}{{|{{\bf{P}}}_{i}^{k}|}_{max}({|{{\bf{P}}}_{i}^{k}|}^{2}+\gamma )}{{\bf{P}}}_{i}^{{k}^{\ast }}({{\rm{\Psi }}}_{i}^{k}-{{\bf{P}}}_{i}^{k}{{\bf{O}}}_{i}^{k})$$

3 $${{\bf{P}}}_{i+1}^{k}={{\bf{P}}}_{i}^{k}-{\beta }^{k}\frac{|{{\bf{O}}}_{i}^{k}|}{{|{{\bf{O}}}_{i}^{k}|}_{max}({|{{\bf{O}}}_{i}^{k}|}^{2}+\gamma )}{{\bf{O}}}_{i}^{{k}^{\ast }}({{\rm{\Psi }}}_{i}^{k}-{{\bf{P}}}_{i}^{k}{{\bf{O}}}_{i}^{k})$$

where ${{\rm{\Psi }}}_{i}^{k}$ is updated sub-spectrum. *k* denotes the iteration number and *i* is the updating image number in this iteration. *α* ^*k*^ and *β* ^*k*^ are the step-size for objective function ${{\bf{O}}}_{i}^{k}$ and pupil function ${{\bf{P}}}_{i}^{k}$. ${\mathcal F} \{.\}$ denotes the discrete Fourier transform while ${ {\mathcal F} }^{-1}\{.\}$ is the inverse discrete Fourier transform. *γ* is a small constant (normally *γ* = 0.01) to avoid divide-by-zero problem when ${|{{\bf{O}}}_{i}^{k}|}^{2}$ or ${|{{\bf{P}}}_{i}^{k}|}^{2}$ is too small. In REFPM, both *α* ^*k*^ and *β* ^*k*^ are halved when no apparent reduction in the error metric is made by the iteration. Moreover, in order to achieve additional improvements of the recovery accuracy, we initial the step-size *α* and *β* as *α* ^0^ = 1, ${\beta }^{0}=\frac{1}{\sqrt{N}}\approx \frac{1}{16}$, where N is the total number of LEDs in the NA of condenser (N = 261).

### Upsampling strategy for FPM

Pixel aliasing problem usually appears in the lens-free on-chip microscopy systems^10, 12, 41, 42^, because the high NA and the large pixel-size of those systems cannot satisfy the Nyquist sampling criterion. Similarly, the pixel aliasing problem could also appear in the FPM platforms. As introduced in ref. 38, the spatial-sampling-ratio of FPM can be presented as ${R}_{cam}=\frac{\lambda }{N{A}_{obj}}\frac{Mag}{2{\rm{\Delta }}{x}_{cam}}$. Considering our REFPM setup (*NA*_*obj*_ = 0.4, *Mag* = 10, Δ*x*_*cam*_ = 6.5 *μm*, *λ* = 633,525,435 nm in R/G/B channels respectively), the pixel aliasing problem would occur in the blue channel (*R*_*cam*_ = 1.22, 1.01, 0.84 in R/G/B channels respectively). Thus we adopt an upsampled FPM method to obtain the expected reconstruction resolution^38^. In the upsampled FPM, we assume that the actual pixel-size of the sensor is reduced by half to guarantee the Nyquist sampling criterion and those low-resolution images are captured when pixel binning is enabled (2 × 2 pixels are combined into one pixel). During the iterative updating procedure, pixel binning processes are complemented computationally to realistically simulate the pixel aliasing problem in the experimental REFPM platform. Since the upsampling strategy for FPM won’t reduce the recovery accuracy even if the spatial sampling criterion is satisfied^38^, we adopt this upsampling strategy in red and green channels too.

### LED positional misalignment correction

As shown in the Fig. 5(a), the R/G/B radiation-emitting semiconductors in one LED are separated with each other at hundreds of micrometers. This little positional uncertainty could significantly affect the recovery accuracy. Therefore, we implement the LED positional misalignment correction method based on simulated annealing and non-linear regression algorithms^37^ for 15 iterations to adjust the positioning errors of all the LED elements. Since the LED array is fixed under the condenser tightly in our REFPM platform, the positional calibration process would be performed only once using the USAF resolution target. Afterwards, the high-resolution images of other samples, such as human blood cell smear, could be reconstructed precisely with no more extra positional correction procedure. Note that the upsampling strategy need to be used in the positional correction process to make sure the positions of the blue LED elements are adjusted accurately. However, the adaptive step-size strategy should not be used in this correction process because if the positional misalignment has not been corrected completely, the decrease of the updating step-size would interfere the positional correction accuracy and result in residual positioning errors. So, in the positional misalignment correction process, we adopt fixed updating step-size *α* = 1, $\beta =\frac{1}{16}$.

### Computation platform used for REFPM

Our reconstructions are performed using MATLAB (Version R2015b, MathWorks, Natick, Massachusetts) on a laptop computer equipped with a 2.60 GHz central processing unit (Intel Core i5-3320M) and 8 GB of random-access memory. In the reconstruction, we divided each full-FOV raw image (2560 × 2160 pixels) into 12 × 10 sub-regions (360 × 360 pixels each), with a 160-pixel overlap on each side of neighboring sub-regions. Each set of images was then processed by the algorithm described above to create a high-resolution reconstruction having both intensity and phase (3600 × 3600 pixels). Finally, all high-resolution reconstructions were combined using the alpha-blending stitching method^13^ to create the full-FOV high-resolution reconstruction. For each 360 × 360 pixels sub-region with an upsampling factor of 10, the processing time of our iterative recovery routine takes ~30 s. The total processing time for the full FOV was nearly 1 h, which could be further reduced by investigating the use of GPU acceleration for our algorithm.

## Electronic supplementary material

Supplementary Video 1:

Supplementary Information
