# Supplementary material for: Resolution-enhanced Fourier ptychographic microscopy based on high-numerical-aperture illuminations
Source: Sci Rep. 2017 Apr 26;7:1187. doi: 10.1038/s41598-017-01346-7 (PMC5430655; doi:10.1038/s41598-017-01346-7)
Supplement: Supplementary file 2 — Supplementary Information [file 41598_2017_1346_MOESM2_ESM.pdf]

Supplementary Information

Resolution-enhanced Fourier ptychographic microscopy based on high-numerical-aperture illuminations

Jiasong Sun[1,2], Chao Zuo[1,2*], Liang Zhang[1,2], and Qian Chen[2*]

[1]Smart Computational Imaging (SCI) Laboratory, Nanjing University of Science and Technology, Nanjing, Jiangsu Province 210094, China

[2]Jiangsu Key Laboratory of Spectral Imaging & Intelligent Sense, Nanjing University of Science and Technology, Nanjing, Jiangsu Province 210094, China

Contents:

A. Comparison Between FPM with Incoherent Microscopy in the Resolution

B. Recovered Phase Maps of Unstained Hela Cells using REFPM

C. Supplementary Video

*Corresponding author: Chao Zuo, Prof,

Nanjing University of Science and Technology

Nanjing, Jiangsu Province 210094, China

Email: zuochao@njust.edu.cn

*Corresponding author: Qian Chen, Prof,

Nanjing University of Science and Technology

Nanjing, Jiangsu Province 210094, China

Email: chenqian@njust.edu.cn

A. Comparison Between FPM with Incoherent Microscopy in the Resolution

Generally speaking, the resolution should be improved with the microscope's NA_{syn} increasing indeed. But it should be emphasized that the final resolution of a microscopy system depends on many factors, such as NA_{syn}, the appearance of the optical transfer function (OTF)[1-3], and imaging pixel size[4,5]. Here, we present some simulation results of different microscopy techniques to explain how these factors affect the final resolution.

In Figure S1, we simulate the reconstructed and captured intensity images of a pure-intensity object using FPM systems and an incoherent microscope and then compare their imaging resolutions. Figures S1(a2)-S1(a4) show the OTFs of an FPM imaging system with 1.6 NA_{syn}, an incoherent microscopy system with 2.55 NA_{syn}, and another FPM system with 2.55 NA_{syn}. Figure S1(b1) is the ideal pure-intensity object, which contains nine resolution elements with different bar widths [listed in Fig. S1(a1)]. Figure S1(c1) presents the line profiles of those resolution elements in Fig. S1(b1). Figures S1(b2)-S1(b4) show the reconstructed or captured images after low pass filtering with their corresponding OTFs. The illumination wavelength in this simulation is 435 nm. Here, Figs. S1(b2)-S1(b4) are captured without pixel sub-sampling, which means Figs. S1(b2)-S1(b4) have the same pixel size (7.2 nm) as Fig. S1(b1). Figures S1(c2)-S1(c4) present the corresponding line profiles of Figs. S1(b2)-S1(b4). As can be seen, the resolutions of Figs. S1(b2)-S1(b4) are 116 nm, 94 nm, and 79 nm, demonstrating the 1.6 NA_{syn} FPM imaging system cannot resolve better than the 2.55 NA_{syn} incoherent microscopy system, which means the resolution could be improved with the NA_{syn} increasing indeed. However, meanwhile, it is shown that the microscopy systems of Fig. S1(b3) and Fig. S1(b4) have a same NA_{syn} but the 2.55 NA_{syn} FPM imaging system achieves a higher resolution. This illustrates that the microscopy resolution cannot be simply determined by the NA_{syn}. The shape of OTF also could be an important influence factor.

Furthermore, we investigate the impact of the imaging pixel size and the pixel sub-sampled images of Figs. S1(b2)-S1(b4) are shown in Figs. S1(d1)-S1(d3) and Figs. S1(f1)-S1(f3) using a pixel-binning algorithm with different imaging pixel size. The intensity images in Figs. S1(d1)-S1(d3) have the same imaging pixel size of 65.0 nm and Figs. S1(f1)-S1(f3) have the same imaging pixel size of 108.3 nm. The line profiles of Figs. S1(d1)-S1(d3) and Figs. S1(f1)-S1(f3) are displayed in Figs. S1(e1)-S1(e3) and Figs. S1(g1)-S1(g3) respectively. When the imaging pixel size increases from 7.2 nm to 65.0 nm, it can be seen that the resolution for each system does not decreases obviously. However, in Figs. S1(g1)-S1(g3) the resolution reduces noticeably when the pixel size is enlarged to 108.3 nm. Especially, comparing with Fig. S1(d2), the 5th element in Fig. S1(f2) becomes totally indistinguishable just because the imaging pixel size is enlarged from 65.0 nm to 108.3 nm. This demonstrates that the imaging pixel size could also play an important role in the resolution of a microscopy system. What's more, since the 5th element in Fig. S1(f3) is still recognizable [also can be recognized in Fig. S1(g3)], it is illustrated that the imaging pixel size is more likely to affect the resolution of an incoherent microscope due to its low contrast.

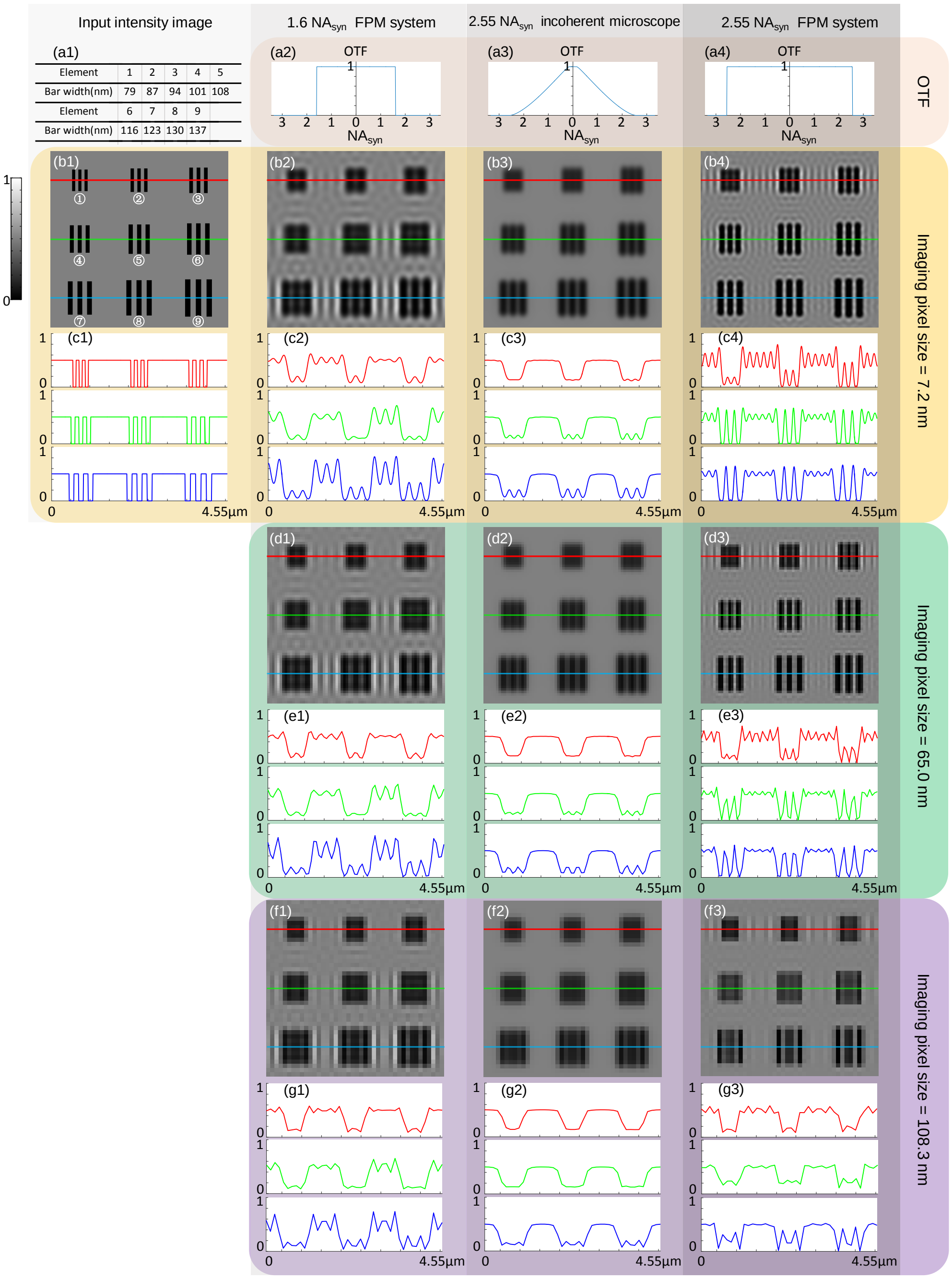

Figure S1 Comparison between FPM with incoherent microscopy in the resolution. (a1) lists the bar widths of nine resolution elements in the ideal intensity image. (a2)-(a4) show the OTF of an 1.6 NA_{syn} FPM system, an 2.55 NA_{syn} incoherent microscope, and an 2.55 NA_{syn} FPM system. (b1) shows the ideal intensity image with a pixel size of 7.2 nm. (b2)-(b4) show the reconstructed or captured images using different systems without pixel sub-sampling. (d1)-(d3) show the pixel sub-sampled images using a pixel-binning method with a pixel size of 65 nm. (f1)-(f3) show the pixel sub-sampled images with a pixel size of 108.3 nm. (c1)-(c4), (e1)-(e3), and (g1)-(g3) show the line profiles of those resolution elements in (b1)-(b4), (d1)-(d3), and (f1)-(f3) respectively.

In our manuscript, the REFPM reconstructs high-resolution images [Figs. 1(e)-1(f) and Figs. 3(d)-3(e)] with a reconstruction pixel size of 65.0 nm and the incoherent microscope records images [Fig. 1(d) and Fig. 3(c)] with an imaging pixel size of 108.3 nm. Comparing Figs. S1(d1) and S1(e1) with Figs. S1(f2) and S1(g2), it can be observed that the resolution of an FPM system with 65.0 nm pixel size is little higher than an incoherent microscope with 108.3 nm pixel size due to their different shapes of OTF and pixel-subsampling rates. This is the reason why 1.6 NA_{syn} coherent imaging can resolve better than 2.55 NA_{syn} incoherent imaging in our manuscript. After all, there is no simple and universal answer to determine the resolution of a microscopy system, which needs specific analyses on a case-by-case basis.

B. Recovered Phase Maps of Unstained Hela Cells using REFPM

Besides phase imaging for stained cell sample (Fig. 4), we also implement our REFPM method for unstained Hela cells with the same experimental parameters and the recovered phase images [Fig. S2(a)-(c)] are displayed below. The whole phase distribution of these unstained Hela cells is illustrated in Fig. S2(a) and the enlarged sub-regions of Fig. S2(a) are shown in Fig. S2(b) and Fig. S2(c). These recovered phase maps demonstrate that REFPM is a practical approach for quantitative phase imaging even if the object's contrast is much weaker.

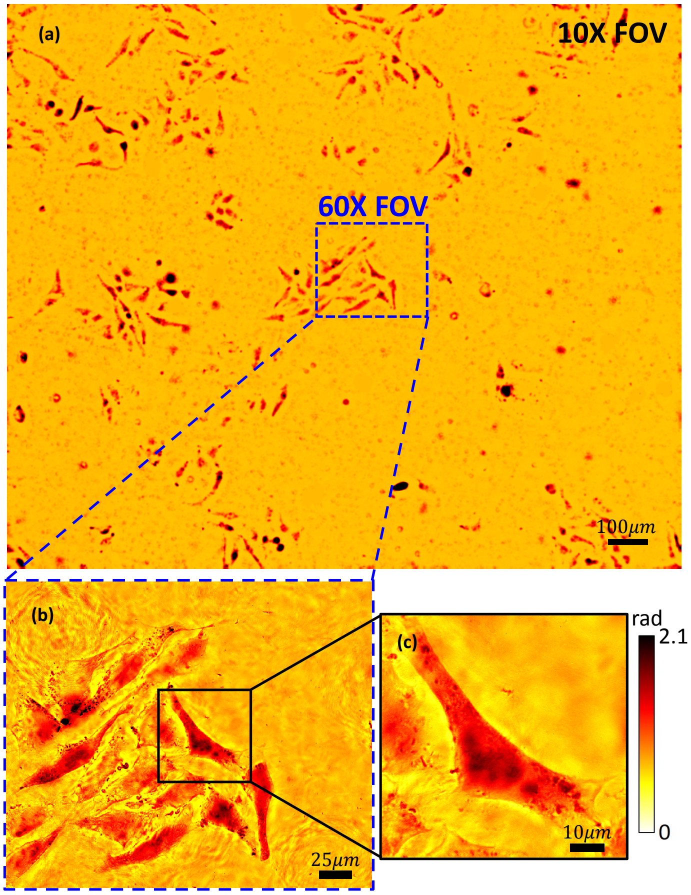

Figure S2 Recovered phase distribution of unstained Hela cells using our REFPM platform. (a) The full FOV of the retrieved phase map using a 10X 0.4 NA objective lens with 1.2 NA illumination. (b)-(c) Enlarged sub-regions of (a).

C. Supplementary Video

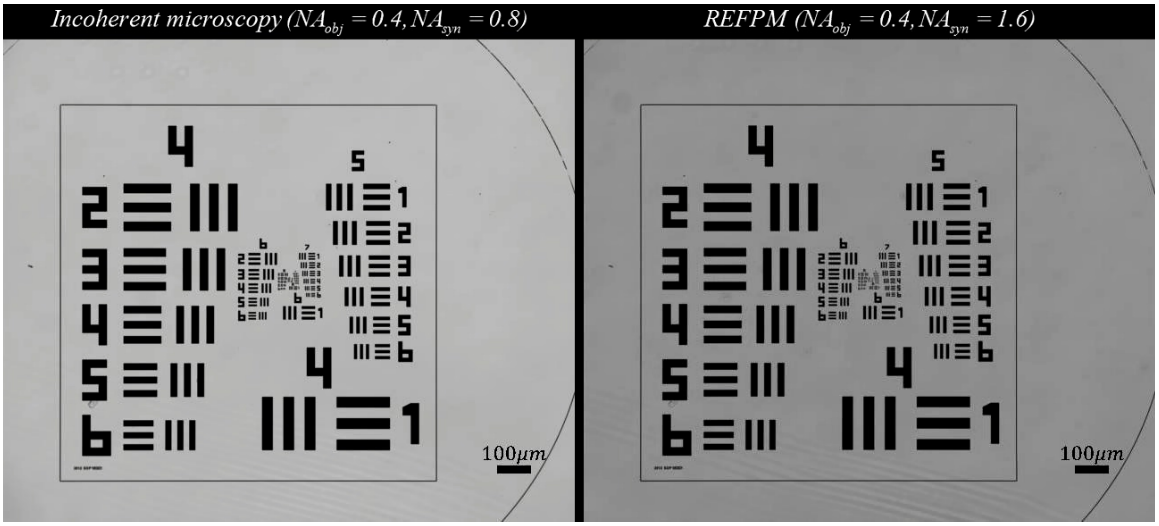

Supplementary Video 1: A zooming video of the full-FOV raw image captured with conventional incoherent microscope ($NA_{obj} = 0.4; NA_{ill} = 0.4; NA_{syn} = 0.8; \lambda = 435nm$) and the reconstructed full-FOV image using REFPM ($NA_{obj} = 0.4; NA_{ill} = 1.2; NA_{syn} = 1.6; \lambda = 435nm$).

References

1. Sheppard, C. J. & Wilson, T. Fourier imaging of phase information in scanning and conventional optical microscopes. *Philosophical Transactions of the Royal Society of London A: Mathematical, Physical and Engineering Sciences* **295**, 513-536 (1980).

2. Rosen, J., Siegel, N. & Brooker, G. Theoretical and experimental demonstration of resolution beyond the Rayleigh limit by FINCH fluorescence microscopic imaging. *Optics Express* **19**, 26249-26268 (2011).

3. Wicker, K. & Heintzmann, R. Resolving a misconception about structured illumination. *Nature Photonics* **8**, 342-344 (2014).

4. Sun, J., Chen, Q., Zhang, Y. & Zuo, C. Sampling criteria for fourier ptychographic microscopy in object space and frequency space. *Optics Express* **24**, 15765–15781 (2016).

5. Bishara, W., Su, T. W., Coskun, A. F. & Ozcan, A. Lensfree on-chip microscopy over a wide field-of-view using pixel super-resolution. *Optics Express* **18**, 11181-11191 (2010).
